# Critically Ill Children in a Swiss Pediatric Emergency Department With an Interdisciplinary Approach: A Prospective Cohort Study

**DOI:** 10.3389/fped.2021.721646

**Published:** 2021-10-11

**Authors:** Leopold Simma, Martin Stocker, Markus Lehner, Lea Wehrli, Franziska Righini-Grunder

**Affiliations:** ^1^Emergency Department, Children's Hospital Lucerne, Lucerne, Switzerland; ^2^Emergency Department, University's Children Hospital Zurich, Zurich, Switzerland; ^3^Neonatal and Pediatric Intensive Care, Children's Hospital Lucerne, Lucerne, Switzerland; ^4^Department of Pediatric Surgery, Children's Hospital Lucerne, Lucerne, Switzerland; ^5^Division of Pediatric Gastroenterology, Department of Pediatrics, Children's Hospital Lucerne, Lucerne, Switzerland

**Keywords:** emergency care, critical ill children, pediatrics—children, resuscitation, critical care, Switzerland/epidemiology, emergency service, pediatric intensive care unit

## Abstract

**Objective:** Delivery of prompt and adequate care for critically ill and injured children presenting to the pediatric emergency department (PED) is paramount for optimal outcomes. Knowledge of the local epidemiology, patient profile, and presentation modes are key for organizational planning, staff education strategy, and optimal care in a PED. Our aim was to analyze the profile of critically ill and injured children admitted to a tertiary, non-academic Swiss PED, to investigate potential risk factors associated with admission to the pediatric intensive care unit (PICU), and the outcomes mortality and PICU admission.

**Methods:** Prospective cohort study of critically ill and injured children presenting to the PED over a two-year period (2018–2019). Inclusion criteria were Australasian triage scale category (ATS) 1, trauma team activation (TTA), medical emergency response (MER) activation, additional critical care consult, and transfer to an outside hospital.

**Results:** Of 42,579 visits during the two-year period, 347 presentations matched the inclusion criteria (0.81%). Leading presentations were central nervous system (CNS) disorders (26.2%), trauma (25.1%), and respiratory emergencies (24.2%). 288 out of 347 cases (83%) arrived during the day or evening with an even distribution over the days of the week. 128 out of 347 (37%) arrived unexpectedly as walk-ins. 233 (67.15%) were ATS category 1. 51% of the cohort was admitted to PICU. Australasian triage scale category 1 was significantly more common in this group (*p* = 0.0001). Infants with respiratory disease had an increased risk of PICU transfer compared to other age groups (OR 4.18 [95%CI 2.46, 7.09] *p* = 0.0001), and this age group presented mainly as walk-in (*p* = 0.0001). Pediatric intensive care unit admissions had a longer hospital stay (4 [2, 8] days vs. 2 [1, 4] days, *p* = 0.0001) compared to other patients. 0.045% of all PED patients had to be transferred out. Three deaths (0.86%) occurred in the PED, 10 patients died in the PICU (2.9%).

**Conclusions:** High acuity presentations in the PED were rare, more likely to be young with CNS disorders, trauma and respiratory diseases. A significant proportion were unexpected walk-in presentations, mainly during day and evening shifts. Low exposure to high-acuity patients highlights the importance of deliberate learning and simulation for all professionals in the PED.

## Introduction

The delivery of emergency medicine is affected by health systems and is highly variable in continental Europe (1). To deliver optimal care, specific knowledge of the local spectrum of disease is essential for training, development of curricula, staffing, and preparation of pediatric emergency departments (PED). In Switzerland, the introduction of pediatric emergency medicine (PEM) in 2014 gradually replaced traditional division into “medical” and “surgical” PEDs. This unification had landmark character and required new organizational structures, training, and culture. A specialized training curriculum for PEM was introduced, and the scope of practice is comparable with PEM in Australia, the United Kingdom, and North America.

The general epidemiological spectrum of PED presentations has been examined in various regions worldwide (2–5). Publications analyzing the epidemiology of critically ill children in the PED are scarce (6–11). One study from Western Switzerland describes a cohort of patients in the resuscitation bay (11). However, we found no recent data for central Europe or Switzerland since the introduction of PEM. With regard to curriculum development, previous publications addressed the problem of higher proportions of low-acuity presentations in children in comparison with adults and resulting issues with critical care skill retention (12, 13). Our goal was to gain insight into the characteristics and epidemiology of children with critical illness and injuries presenting to a tertiary, non-academic PED in Switzerland, and whether the spectrum differs from other high-income countries. Furthermore, we investigated potential risk factors associated with transfer to the pediatric intensive care unit (PICU). This has important implications for patient safety and impacts training, design of infrastructure, and processes within institutions.

## Methods

Prospective cohort study with retrospective analysis of critically ill children presenting to the PED of our institution over a 2-year period. The study was approved by the regional Ethics Committee (EKNZ 2020-00155), which gave consent to collect individual data of all children presenting to our PED between January 1, 2018 and December 31, 2019.

### Setting

The setting is a tertiary pediatric PED with an annual census of 21,000 presentations and designated national level-1 trauma center. We serve an urban and rural population of approximately 810,000 inhabitants in a geographically confined area and treat patients under 16 years as well as older patients with chronic pediatric conditions. The emergency department is staffed with PEM-trained pediatricians from 08:00 to 23:00, and general pediatricians and subspecialty consultants are on-call at night from home. Pediatric intensive care attendings are in-house for 24 h. Senior pediatric surgeons are in-house during business hours and on call out of hours (17:00–8:00, weekends and public holidays). Pediatric trainees and pediatric surgery residents with postgraduate experience from 0 to 6 years are assigned to six shifts per 24 h. Most Swiss pediatric hospitals use the five-level Australasian triage scale (ATS) as the standard patent triage tool (14). Australasian triage scale category 1 requires immediate simultaneous assessment and treatment of a life-threatening condition (15). Major trauma patients are treated in a trauma bay shared with the adult hospital on the same campus and ensures rapid access to computed tomography and magnetic resonance imaging. The pediatric trauma team comprises senior staff of pediatric surgery, PED, PICU, and pediatric anesthesia.

A medical emergency response (MER) team consisting of senior PICU and anesthesia staff can be activated to assist PED staff with non-trauma resuscitations (drownings, intoxications, cardiac arrests, etc.). Staffing of teams is shown in Supplementary Table 1. Both immediate response teams are activated by a phone conference call and follow advanced trauma life support (ATLS^®^) and pediatric advanced life support (PALS^®^) guidelines. The trauma procedure of the institution was customized based on previous publications (16, 17).

### Inclusion Criteria and Data Collection

We used prospectively collected data from a dedicated emergency department registry ranging from January 2018 to December 2019 of pediatric patients aged from 0 to 18 years. These data were analyzed retrospectively.

Inclusion criteria for definition as critically ill or injured were one or more of the following: ATS category 1, trauma team activation (TTA), MER, additional critical care consult by senior PICU staff and/or anesthesia staff, and transfer to an outside hospital. There were no additional exclusion criteria.

We collected patient age, gender, ATS, time, and day of presentation (morning 08:00–16:00, evening 16:00–23:00, and night 23:00–08:00), mode of arrival, diagnosis, diagnosis category according to the primary diagnosis, comorbidity, consult by senior PICU staff, and/or anesthesia staff, outcome [disposition (inpatient ward, PICU, external transfer, and discharge from PED), length of hospital stay, and death].

For data extraction, the electronic medical record, emergency department information system, and electronic TTA-log were used. Data were collected in an emergency department registry, and patients were followed until discharge from the hospital.

### Data Analysis and Statistics

Descriptive data are presented as median and interquartiles (Q1, Q3) for continuous variables after checking for Gaussian distribution and as frequency (%) for categorical variables. Demographics and characteristics of patient groups were compared by using the Pearson's χ^2^-test or Fisher's exact test for categorical variables and Wilcoxon rank sum test for continuous variables.

Multivariable regression analysis was performed to assess potential risk factors associated with PICU transfer using a logistic regression model. Clinically important variables, or variables with significance at *p*-value ≤ 0.2 on univariate analysis testing were included [diagnosis category, type of presentation (trauma vs. non-trauma), mode and time of initial presentation at the PED (walk-in vs. non-walk-in; morning, evening vs. night shift of presentation)]. Interactions were tested before setting up the definitive model. The final model was adjusted for age at the time of presentation. For statistical testing purposes, diagnosis categories accounting for <5% of the total were summed under “miscellaneous.” All analyses were performed using the SAS software, version 9.3 (SAS Institute, Cary, NC, USA); all statistical tests were two-sided. Values of *p* ≤ 0.05 were considered statistically significant for all analyses.

## Results

**Figure 1:**
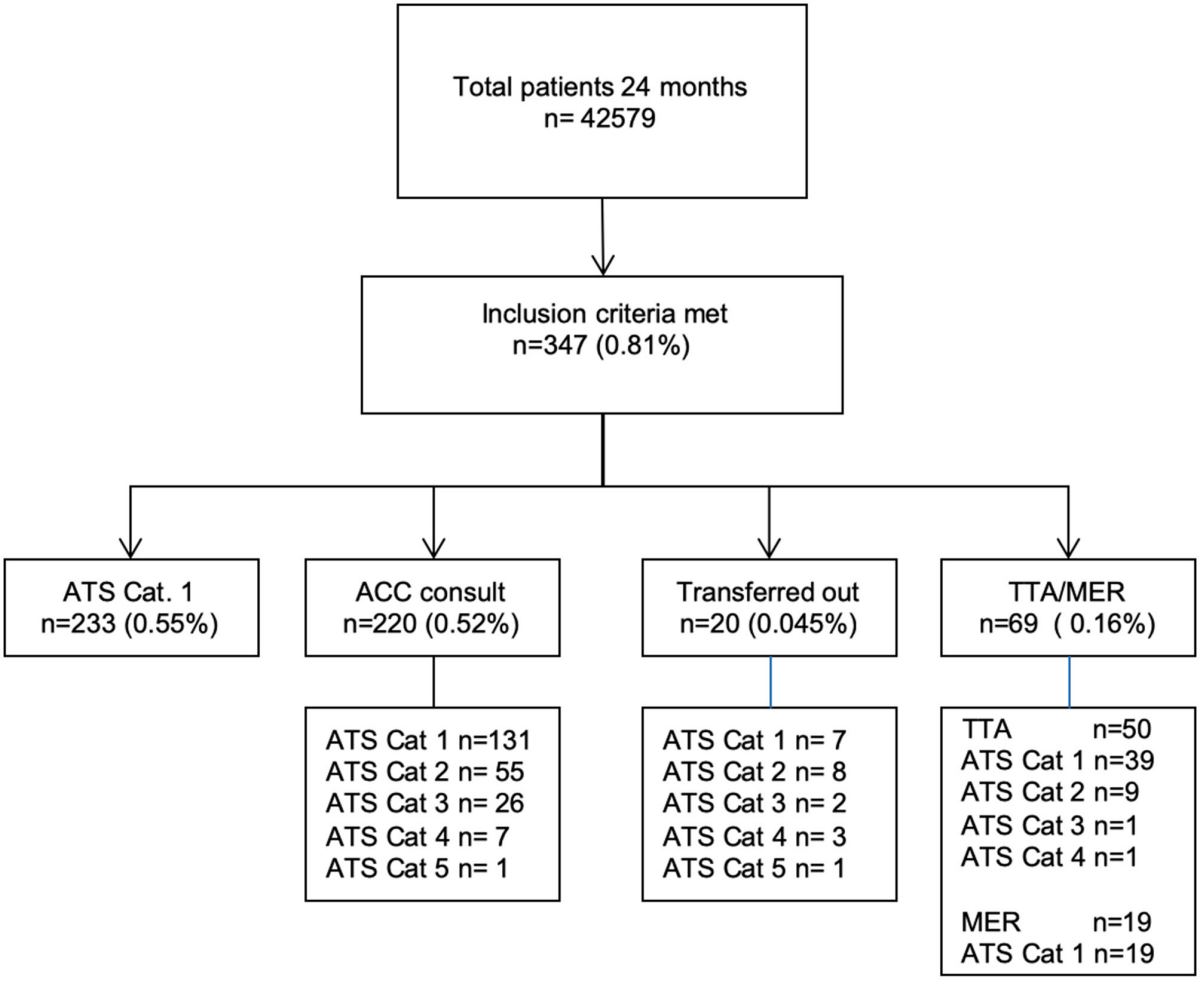
Patient inclusion and triage categories. ATS, Australasian triage scale; ACC consult, additional critical care consult by pediatric intensive care staff and/or anesthesia staff; TTA, trauma team activation; MER, medical emergency response.

A total of 42,579 visits in the PED were recorded during the two-year period, with an overall admission rate of 13.5%. 347 presentations matched the inclusion criteria (0.81%). Figure 1 shows the reasons for inclusion with the corresponding ATS.

### Patients Characteristics and Epidemiology

Patient characteristics are depicted in Table 1. The most frequent diagnosis categories were central nervous system (CNS) disorders, trauma, and respiratory disease. Categories with seasonal variation were trauma with a surge in the warm months (April–October) and respiratory disease in in the cold months (*p* = 0.0006). A detailed breakdown of the diagnosis categories and diagnoses are shown in Supplementary Table 2.

**Table 1:** Characteristics of patients—sex, age, ATS category, disposition (*n* = 347).

	***n***	**Percent (%)**
**Sex**		
Female	138	39.77
Male	209	60.23
**Age**		
Median, IQR (year)	3.0 [0, 9]	
Age range	2 days to 18 years	
**Age groups**		
<1 month	21	6.05
0–12 months	91	26.22
1–4 years	110	31.70
5–12 years	87	25.07
13–18 years	59	17.00
**Australasian triage scale**		
Category 1	233	67.15
Category 2	72	20.75
Category 3	28	8.07
Category 4	13	3.75
Category 5	1	0.29
**Mode of arrival**		
Ambulance	144	41.50
Walk-in	128	36.89
Referral by primary care^*^	40	11.53
HEMS	35	10.09
**Time of arrival**		
08:00–16:00	156	45
16:00–23:00	132	38
23:00–08:00	59	17
**Day of arrival**		
Weekday	246	70.9
Weekend	101	29.1
**Disposition**		
PICU	180	51.87
- ATS Category 1	93	51.67
Operating room (OR)	31	8.96
- PICU *via* OR	19	5.48
- Inpatient *via* OR	11	3.17
- OR and transfer	1	0.29
In-patient ward	131	37.75
Discharge home	13	3.75
Transfer to other facility	20	5.48
Death	3	0.86
**Diagnosis categories**		
CNS	91	26.22
Trauma	87	25.07
Respiratory	83	23.92
Cardiovascular	19	5.48
Metabolic	15	4.32
Gastrointestinal	12	3.46
Intoxications	12	3.46
Infections	8	2.31
Neuropsychiatric	7	2.02
ENT	6	1.73
Hematologic	3	0.86
Multisystem	2	0.58
Oncologic	2	0.58
**Reasons for transfers to other facilities**	20	5.48
Congenital heart disease	6	1.73
Mental health problem	5	1.44
Transition to adult care	5	1.44
Esophageal varices bleed	1	0.29
Severe scald burn	1	0.29
STEMI (18 years, CF)	1	0.29
Bed block	1	0.29

*HEMS, helicopter emergency services; OR, operating room; PICU, pediatric intensive care unit; CNS, central nervous system; ENT, ear–nose–throat emergency*;

* *primary care, pediatrician or general practitioner (GP); STEMI, ST elevation myocardial infarction; CF, cystic fibrosis. Data are presented as n (%) for categorical variables and as median (IQR q1, q3) for continuous variables*.

Patients in critical condition presented mainly during day and evening shifts (83%); nightly presentations were relatively rare (17%). There was an even distribution of visits during the week vs. the weekend [OR 1.09 (95% CI 0.66, 1.78); *p* = 0.75], and no difference in subsequent PICU transfer was seen (*p* = 0.64). The subgroup of ATS category 1 patients that was stabilized in ED and admitted to an inpatient unit (*n* = 132) had a higher median age of 4 years [(1, 9), *p* = 0.02] than PICU admissions. In these stabilized patients, leading diagnosis groups were also predominantly acute CNS disorders (*n* = 44, 33.3%), trauma (*n* = 40, 30.3%), and respiratory disease (*n* = 28, 21.2%).

### Mode of Presentation: Walk in vs. Arrival With Medicalized Transport

**Figure 2:**
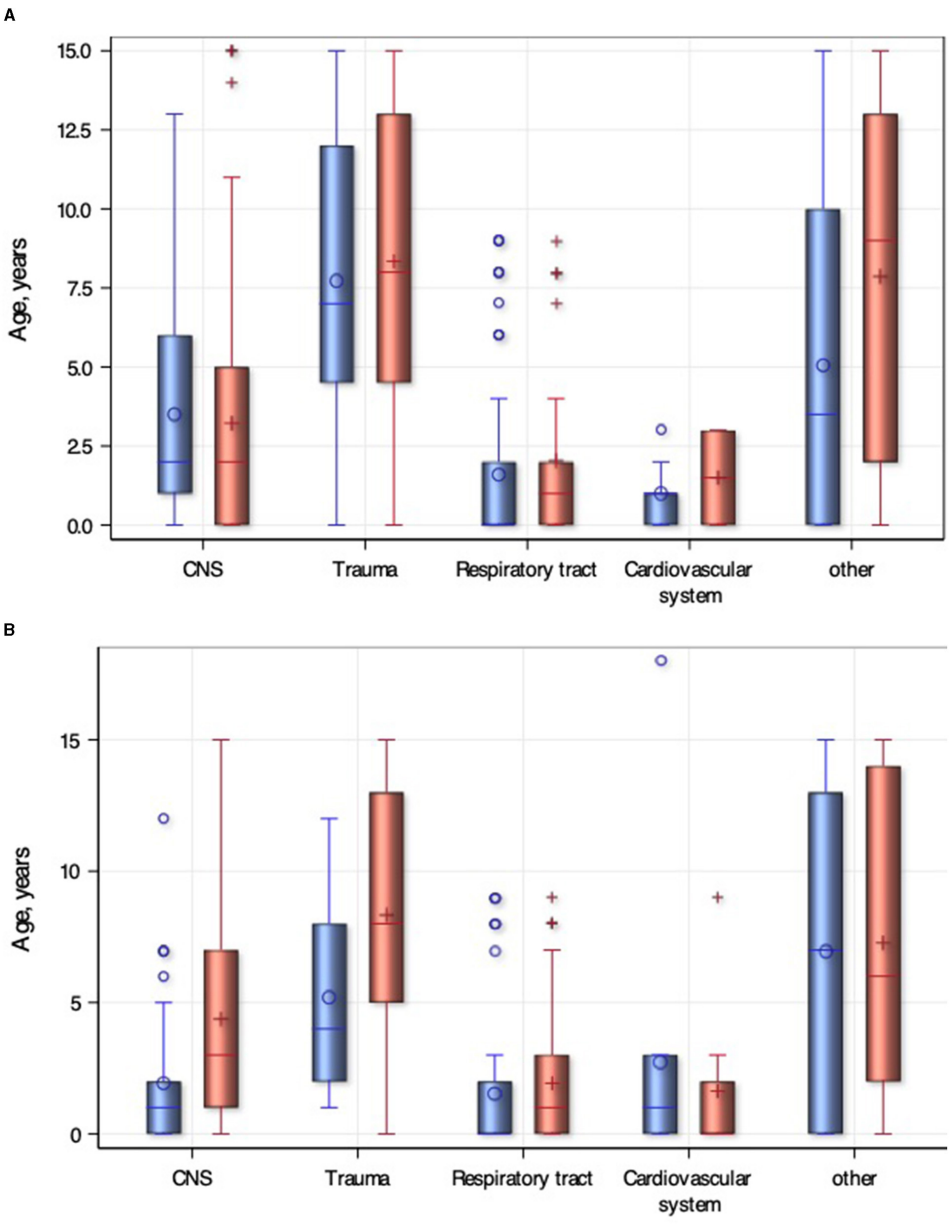
Profiles of pediatric intensive care unit (PICU) admissions and transport modes. **(A)** PICU Transfers by diagnosis group and age. Blue box plots - PICU admission, Red box plot - inpatient (ward) admission. **(B)** Walk-in versus medicalized transport, Blue box plots - Walk-in, Red box plot - medicalized transport.

An overview of the presentation mode is shown in Table 1. Children younger than 1 year of age presented to the PED more frequently as walk-ins, compared with older age groups (*p* < 0.0001). This group had two- to four-fold chance of walk-in presentation compared with all the other age groups, using simple logistic regression [1- to 4-year OR 1.8 (95% CI 1.01, 3.23), 4- to 10-year OR 3.97 (95% CI 2.07, 7.57), and >10 years OR 2.97 (95% CI 1.54, 5.76)]. Patients with respiratory disease presented predominantly by walk-in [OR 1.89 (95% CI 1.15, 3.12); *p* = 0.01]. Figure 2B shows the presentation mode grouped by age and diagnosis category.

### Immediate Response Activation

There were 69 immediate response activations of which 50 were TTAs. Patients with immediate response activation (MER or TTA) had a significantly higher median age [6 years (2, 12) vs. 2 years (0,8, *p* = 0.0002)] were more likely to arrive by medicalized transport [reverse OR 0.08 (0.03, 0.22), *p* < 0.0001], be trauma patients [reverse OR 0.12 (0.07, 0.23), *p* < 0.0001], and more likely to have an intensivist present at arrival [OR 14.48 (5.14, 30.8), *p* < 0.0001]. However, there was no significant association between admission to PICU and TTA/MER [OR 0.76 (0.44, 1.3) *p* = 0.31]. No difference in immediate response activation by shift was found by logistic regression, but total numbers were lower at night.

### Potential Risk Factors Associated With Pediatric Intensive Care Unit Transfer

Of the critically ill or injured patients, 51% were admitted to the PICU. Pediatric intensive care unit admissions vs. inpatient admissions were compared by univariate analysis (Table 2). The proportion of PICU admissions was significantly more common with ATS category 1 (51.7%, *n* = 93) vs. all other ATS categories (48.3%, *n* = 87) (*p* < 0.0001). The most common diagnosis groups for ED presentations remained unchanged in PICU-transferred patients, with respiratory causes being the leading reason for PICU transfer [OR 1.91 (95% CI 1.13, 3.22); *p* = 0.02]. Children transferred to the PICU were younger [2 years (0, 7) vs. 4 years (1, 9); *p* = 0.02] and were less likely to be female [OR 0.55 (95% CI 0.35, 0.86); *p* = 0.008]. In this cohort, infants had an increased risk of PICU transfer compared with 4 to 10-year olds [OR 1.87 (1.01, 3.44)], in particular, infants with respiratory disease [OR 4.18 (95% CI 2.46, 7.09) *p* ≤ 0.0001] compared with all older patients. High-flow nasal cannula (HFNC) was available from December 2018 and was initiated in 12 patients in the PED. All required transfer to the PICU. Three patients in the sample deteriorated with low-flow oxygen on the ward requiring HFNC. Out of these three patients, one subsequently met the criteria for PICU admission. Figure 2A shows the age distribution and diagnosis groups in patients transferred to PICU. Multivariable logistic regression analysis showed that male gender [OR 1.81 (95% CI 1.11, 2.92)], PED presentations during morning shifts [OR 2.66 (95% CI 1.32, 5.34)], and evening shifts [OR 2.82 (95%CI 1.39, 5.73)] compared with night shifts were potential risk factors for a PICU admission (Table 3).

**Table 2:** Analysis of PICU admissions—univariate analysis.

	**Transfer** **to PICU**,	**No transfer** **to PICU**,	**OR**	***p*-Value**
	***n* = 180** ^*^	***n* = 144** ^*^		
**Age, median, years**	2 (0, 7)	4 (1, 9)	–	0.02
**Gender**				
Female	59 (33)	68 (47)		
Male	121 (67)	76 (53)	0.55 (0.35, 0.86)	0.008
**Mode of arrival**				
Walk-in	66 (37)	52 (36)		
Non walk-in	114 (63)	92 (64)	1.02 (0.65, 1.62)	0.92
**Presence of PICU team**				
Yes	160 (89)	43 (30)		
No	20 (11)	101 (70)	18.79 (10.46, 33.76)	<0.0001
**Admission**				
Weekend	52 (29)	45 (31)		
Weekday	128 (71)	99 (69)	0.89 (0.55, 1.44)	0.64
**Admission daytime**				
Morning shift	85 (47)	59 (41)		
Evening shift	74 (41)	34 (24)		
Night shift	21 (12)	51 (35)	–	0.017
**Admission season**				
Cold months (November–March)	95 (53)	77 (53)		
Warm months (April–October)	85 (47)	67 (47)	0.97 (0.63, 1.51)	0.9
**Anesthesia involvement**				
Yes	45 (25)	35 (24)	1.04 (0.62, 1.73)	0.89
No	135 (75)	109 (76)		
**Diagnosis category**				
CNS	40 (22)	49 (34)		
Trauma	40 (22)	44 (31)		
Respiratory	55 (31)	27 (19)		
Cardiovascular	9 (5)	2 (1)		
Other	36 (20)	22 (15)	–	0.0054
**Outcome**				
**Death** ^*^				
Yes	10 (5)	0 (0)		
No	180 (95)	144 (100)	–	–
**Need for surgical intervention**				
Yes	19 (11)	11 (8)		
No	160 (89)	133 (92)	1.44 (0.66, 3.12)	0.36
**LOHS, median, days**	4 [2, 8]	2 [1, 4]	–	<0.001

* *Excluded 23 of total 347 (3 deaths in ED, 20 transfer to other hospitals). PICU, pediatric intensive care; CNS, central nervous system; LOHS, length of hospital stay. Data are presented as n (%) for categorical variables and median (IQR q1, q3) for continuous variables*.

**Table 3:** Multivariable logistic regression analysis: factors associated with PICU admission; analysis of maximum likelihood estimates and OR estimates.

	**OR (95% CI)**	**Estimates (95% CI)**	***p*-Value of estimates**
**Age**	0.96 (0.91, 1.02)	−0.04 (−0.1, 0.02)	0.16
**Gender (M vs. F)**	1.81 (1.11, 2.92)	0.29 (0.05, 0.54)	0.02
**Diagnosis category**			
CNS	0.4 (0.19, 0.84)	−0.46 (−0.84, −0.09)	0.02
Trauma	0.41 (0.19, 0.87)	−0.45 (−0.83, −0.07)	0.02
Respiratory	0.94 (0.43, 2.06)	−0.03 (−0.42, 0.36)	0.87
Cardiovascular	2.62 (0.48, 14.34)	0.48 (−0.37, 1.33)	0.27
Walk-in vs. non-walk-in^*^ **presentation**	0.79 (0.47, 1.35)	−0.12 (−0.38, 0.15)	0.39
**Presentation timing**			
Morning vs. night	2.66 (1.32, 5.34)	0.31 (−0.03, 0.64)	0.07
Afternoon vs. night	2.82 (1.39, 5.73)	0.37 (0.02, 0.71)	0.04

* *All other modes of arrival: medicalized transport by road/air, primary care referral*.

### Outcome

Pediatric intensive care unit admissions had a longer hospital stay [4 (2,8) days vs. 2 (1,4) days, *p* < 0.0001] compared with ward admissions. Outcomes of PICU admissions were as follows: 10 died and 170 survived. Of the survivors, eight required further treatment at other centers, psychiatry, or rehabilitation. Thirty-one patients required an intervention in the operating room (surgery or bronchoscopy), and more than half of these (*n* = 19) were consecutively admitted to the PICU. Three patients died in PED (all ATS category 1; two out-of-hospital cardiac arrests, one major trauma). Thirteen patients were discharged home from the PED. Four were trauma activations (two each arrived by ambulance and helicopter, respectively), the remainder were mostly seizures, often with known epilepsy. All patients admitted to inpatient units survived.

## Discussion

Critically ill patients comprise a small fraction of the PED case load in our geographically confined region. Leading presentations were CNS disorders, trauma, and respiratory emergencies. Respiratory emergencies also had the highest risk for PICU transfer. Overall, about 50% of the patients triaged as “immediate” (ATS category 1) were admitted to the PICU. Most of the critically ill patients in the cohort were very young. Critically ill adolescents were an uncommon scenario. Death in the PED was rare and has been reported to be a rare occurrence in PEDs in high-income countries (10). In our PED, three deaths (0.86% of the cohort) occurred, and 10 patients died in the PICU (2.9%). Only 0.045% of all PED patients had to be transferred out of our children's hospital, and the majority received further care locally in either adult or psychiatric facilities. To our knowledge, this is the first study to examine critically ill and injured pediatric patients and their risk factors for PICU admission in an interdisciplinary PED since the introduction of PEM in Switzerland.

Epidemiological data examining critical emergency presentations to children's hospitals is scarce. In our cohort, only 0.81% of the 42,579 visits met the inclusion criteria and qualified as critical by our definition. The existing data show comparable numbers of critical PED visits. International reports range from 0.7% in the UK and Korea (18, 19), 0.6% in southern France (6) and Australasia (20), 0.45% in western Switzerland (11), and 0.3% in western Canada (21). Central nervous system disorders were the most frequent presenting problem in our sample, which was also reported in cohorts examined in the US (10), western Switzerland (11), and in the UK (18). Many of these were seizures with clear-cut treatment algorithms; the remainder were presentations with altered mental status (non-intoxications).

At one Canadian Center, respiratory illness was the leading category for PICU admission over a 2-year period (21) and, in a US national cohort, the most frequent serious pediatric emergency condition (22). Higher risk of PICU admission in respiratory disease may be the result of age profile and disease burden. About halfway through the study period, HFNC became available in the PED. Of the few patients that were initiated on HFNC in the PED, none met the criteria for ward admission. With cautious interpretation, this intervention did not appear to impact PICU admissions in the introductory phase of HFNC in the PED. Cardiovascular emergencies appeared to be as infrequent as in other high-income countries (7, 9, 10, 21). Studies reporting higher incidence of cardiovascular causes included all subclasses of shock or “near-miss” SIDS into this category; also, shock was exceedingly rare in the period observed (4, 6).

Numbers of trauma patients may differ at similar-sized centers without level one status. It is well-known that the criteria for TTA vary among institutions (23), and different activation thresholds may result in considerable variation in the numbers of trauma calls. However, even for a designated level 1 center, the case load only adds up to two TTA per month, making major trauma an infrequent scenario. This finding is compatible with national registry data from the UK and Germany, where pediatric major trauma accounts for <5% of total trauma burden (24, 25).

A study from western Switzerland investigated life-threatening emergencies in the resuscitation bay (11). This cohort had different inclusion criteria, examining only direct admissions to the resuscitation bay and was using the National Advisory Committee of Aeronautics (NACA) score as a surrogate for a triage scale. The NACA score limits comparability of clinical severity, as it is intended for preclinical use. The PED setting differs in separation into surgical and medical teams in a pre-PEM era in Switzerland. The overall PICU admission rate was similar (41.2%). The ranking of diagnosis categories is very similar to our cohort, but reported mortality rates were significantly higher (7.2 vs. 0.9%). This may be due to selection bias as it is a center with pediatric cardiac surgery and burns unit.

Assessment of patients is a challenge both in-hospital and prehospital. Scarce prehospital information may result in suboptimal preparation and anticipation. Inaccurate prehospital information can lead to low anesthesia staff involvement and high risk of PICU admission (Table 2). In contrast, low-quality information also resulted in discharge home from ED after TTA (Table 1). The issue of low-quality prehospital communication has been investigated leading to recommendations for standardizing handovers (26, 27). However, an important proportion of the cohort presented without prior notice. Nearly 37% of critical patients presented directly to triage, thus, allowing no lead time for preparation (Figure 2B; Table 1). On the other hand, in-hospital triage assessments can vary and represent only a snapshot of the condition of the patient (28). Patients may deteriorate during the stay in the PED. Both could be represented by 48% of PICU admissions being non-ATS category 1 patients (Table 1). This finding highlights the need for high-quality nursing and adequate staffing at the entry point (the triage area) and also in non-high-dependency areas of the PED. All these factors underscore the importance of a 24/7 interdisciplinary setting with PEM physicians, general pediatricians, and easily accessible senior support of pediatric anesthesia and PICU.

### Impact on Organization and Training

Crowding of the PED on weekends with a constant number of critical patients can have implications on patient safety as published by Michelson et al. (29). This should be factored in, when planning staffing and institutional response algorithms for medical or trauma emergencies. In our cohort acute neurologic problems, trauma and respiratory disease was frequent. However, with an average of two trauma activations per month and low numbers of cardiovascular problems, maintenance of skills for the individual senior medical staff member in regular practice is difficult. Various authors have reported lack of exposure to critical procedures (21, 30). This has major implications for trainees and staff physicians alike. Li et al. recently described low pediatric critical case exposure in emergency medicine trainees in the US (31). Our findings are similar to previous reports in terms of low exposure to high-acuity patients, which is a concern even at high-volume centers (12, 30). This has major implications for acquisition and retention of critical skills with an overall lower acuity in the PED. As a consequence, continuous efforts for training opportunities need to be made. This underscores the importance and for simulation-based training (13, 32). At our hospital, a simulation program was implemented a few years ago (33), but a national survey reported underuse and low dissemination in Swiss pediatric hospitals (34). Our results suggest that structured trauma education should be high on the priority list for pediatric emergency providers, as this is beyond the scope of basic pediatric training in Switzerland. Cardiac emergencies remain a high-stake, low-opportunity scenario. A summary of relevant skills and interventions is shown in Supplementary Table 3. Ongoing efforts are needed to maintain knowledge of the relevant algorithms and smooth delivery of time-critical procedures. This data will help guide planning and preparation for the care of critically ill or injured pediatric patients. Financial support for running continuous simulation programs encompassing all seniority levels must be allocated to ensure the best care and outcomes.

## Limitations

Our data are from a single center in a high-income country, which, per definition, limits generalizability. It is also possible that it may not be nationally representative. However, as we serve a geographically confined area with a mixed urban and rural population, the risk of bypassing our institution or funneling off to other pediatric centers is minimal and reduces referral bias influence. We believe that our results are likely to approximate the general pediatric emergency care practice.

Human factors also contribute to the allocation of patients into triage categories, which leads to over- and undertriage, and previous publications have addressed this issue regarding interrater reliability (28, 35). Australasian triage scale category 2 was not used as a primary inclusion criterion, as it often used to expedite management of patients needing rapid attention (e.g., severe pain, fever in a neonate, testicular complaint, etc.), but these may not be critically ill *per se*. However, by adding PICU admissions in the analysis, the impact of undertriage is attenuated, and clinical deterioration during the PED visit is captured as well. Admission criteria to PICU may depend on multiple factors and may be not always be clear cut (details shown in Supplementary Table 4). One strength of this study is its prospective data collection with a multilevel approach, which enabled patient identification, that may have been missed in one data source to be identified by another.

## Conclusions

High acuity presentations only make up a fraction of the total patient load in the PED. Critical patients are more likely to be young presenting with CNS disorders (mostly seizures or altered mental status), major trauma, and respiratory diseases. The main risk factors associated with PICU admission were young age and respiratory disease. We are convinced and we believe that PEM with an interdisciplinary approach and readily available support by anesthesia, pediatric surgery, and senior PICU staff in the PED is a key factor for favorable outcomes. Low exposure to high-acuity patients highlights the importance of deliberate learning and simulation for all professionals in the PED. Our findings translate into high priority of sustained efforts to maintain capability in managing critically ill and injured children in the PED.

## Data Availability Statement

The datasets presented in this article are not publicly available as this is not in the scope of the ethical approval for the use of this data. Requests to access the datasets should be directed to leopold.simma@kispi.uzh.ch.

## Ethics Statement

The studies involving human participants were reviewed and approved by Ethikkommission Nordwest- und Zentralschweiz (EKNZ), Basel. Use of the data for this study was approved by the responsible ethics committee (EKNZ 2020-00155). Informed consent for publication of anonymised patient data was waived for this study by the ethics comittee.

## Author Contributions

LS conceived the study, collected the data, compiled descriptive statistics, and wrote the initial draft. FR-G performed the statistical analysis. FR-G, ML, MS, LS, and LW interpreted the statistical data. All authors made significant contributions to the final draft.

## Conflict of Interest

The authors declare that the research was conducted in the absence of any commercial or financial relationships that could be construed as a potential conflict of interest.

## Publisher's Note

All claims expressed in this article are solely those of the authors and do not necessarily represent those of their affiliated organizations, or those of the publisher, the editors and the reviewers. Any product that may be evaluated in this article, or claim that may be made by its manufacturer, is not guaranteed or endorsed by the publisher.

## Abbreviations

ATS: Australasian triage scale
CNS: central nervous system
EDIS: emergency department information system
ENT: ear, nosethroat
HEMS: helicopter emergency medical service
MER: medical emergency response
NACA: National Advisory Committee of Aeronautics
OR: operating room
PED: pediatric emergency department
PEM: pediatric emergency medicine
PICU: pediatric intensive care unit
SIDS: sudden infant death syndrome
TTA: trauma team activation.
