# Supplementary material for: Critically Ill Children in a Swiss Pediatric Emergency Department With an Interdisciplinary Approach: A Prospective Cohort Study
Source: Front Pediatr. 2021 Oct 11;9:721646. doi: 10.3389/fped.2021.721646 (PMC8544259; doi:10.3389/fped.2021.721646)
Supplement: Supplementary file 1 [file Data_Sheet_1.pdf]

**Supplemental Table 1 - Resuscitation team composition**	
**Trauma resuscitation (TTA)**	
Team leader	Senior PED physician, senior PICU physician, or senior pediatric surgeon
Airway	Senior anesthetist, anesthesia resident, and nurse
Circulation	Senior PICU physician, PICU resident, and nurse
Assessment doctor	Pediatric surgeon (resident or senior)
Scribe	PED resident, adult ED nurse
Optional: Team leader support	Adult trauma surgeon
Optional: subspecialty support	Neurosurgery, ENT
**Non-trauma resuscitation (MER)**	
Team leader	Senior PED physician or senior PICU physician
Airway	Senior anesthetist, anesthesia resident, and nurse
Circulation	Senior PICU physician or senior PED physician, PICU/PED resident, and nurse
Optional: assessment doctor	Pediatric PED resident/PICU resident if available
Scribe	PED resident, adult ED nurse
Optional: subspecialty support	Cardiology, infectious diseases, nephrology, gastroenterology, respiratory medicine, diabetes and endocrinology

TTA- trauma team activation, MER – medical emergency response, PED – pediatric emergency department, PICU – pediatric intensive care unit, ED – emergency department, ENT – otorhinolaryngology
See: https://www.kispi-wiki.ch/interdisziplinare-notfallstation-ins/schockraumkonzept-kispiluks/schockraum-konzept-kind (in German, Version January 2020, accessed 21.08.21)
