# Supplementary material for: Critically Ill Children in a Swiss Pediatric Emergency Department With an Interdisciplinary Approach: A Prospective Cohort Study
Source: Front Pediatr. 2021 Oct 11;9:721646. doi: 10.3389/fped.2021.721646 (PMC8544259; doi:10.3389/fped.2021.721646)
Supplement: Supplementary file 2 [file Data_Sheet_2.pdf]

Supplemental Table 2 - Breakdown of diagnoses by organ system and comorbidity		
**Neurologic**	**n= 91 / 347**	**26.2%**
Status epilepticus	33	36.3%
Seizures	36	39.6%
Shunt dysfunction/hydrocephalus/ increased intracranial pressure	9	9.9%
Altered mental status	6	6.6%
Non-traumatic brain hemorrhage	2	2.1%
Non-traumatic spinal lesion	1	1.1%
Suspected stroke	1	1.1%
Severe myoclonia	1	1.1%
Cerebral thrombosis	1	1.1%
Intracranial abscess	1	1.1%
Comorbid patients	21	23.1%
**Trauma**	**n= 87 / 347**	**25.1%**
Trauma team activation*	50	57.5%
Trauma w/o trauma team activation	37	42.5%
- Cat 1	19	51.4%
- Cat 2	10	27.0%
- Cat 3	5	13.5%
- Cat 4	2	5.4%
- Cat 5	1	2.7%
Comorbid patients	1	0.11%
**Respiratory**	**n= 83 / 347**	**23.9%**
Lower airway disease[1]	53	63.9%
Upper airway disease[2]	11	13.3%
Apnoea[3]	14	16.8%
Foreign body / aspiration	5	6%
Comorbid patients	11	13.3%
**Cardiovascular**	**n= 19 / 347**	**5.48%**
Arrhythmia	10	52.6%
Congenital heart disease	5	26.3%
Cardiac arrest	3	15.7%
Acute coronary syndrome	1	5.3%
Comorbid patients	8	42.1%
**Miscellaneous**	**n= 67 / 347**	**19.31%**
Comorbid patients	17	25.4%

Includes categories metabolic, gastrointestinal, intoxications, infections, neuropsychiatric, ENT, hematologic, multisystem, oncologic;
Frequent problems: diabetes mellitus (hypoglycemia, ketoacidosis), severe metabolic disorders, severe electrolyte disturbances, alcohol intoxications, other intoxications, bleeding disorders, sepsis, systemic infections, inborn errors of metabolism, acute leukemia, hemolysis, rhabdomyolysis, toxic shock syndrome, acute abdomen, psychiatric (suicidal ideation, dissociative disorders, aggression, self-harm)

* breakdown of triage categories is shown in patient enrolment pathway (Figure 1)
[1] includes bronchiolitis, bronchitis, asthma, pneumonia, viral pneumonitis,
[2] includes laryngotracheitis (croup), upper respiratory infection w/ respiratory distress
[3] includes apnea, apparent life-threatening events (history of apnea and CPR by caregiver), pertussis, exacerbated disorders of respiratory drive presenting with apnea
