# Supplementary material for: Critically Ill Children in a Swiss Pediatric Emergency Department With an Interdisciplinary Approach: A Prospective Cohort Study
Source: Front Pediatr. 2021 Oct 11;9:721646. doi: 10.3389/fped.2021.721646 (PMC8544259; doi:10.3389/fped.2021.721646)
Supplement: Supplementary file 3 [file Data_Sheet_3.pdf]

Supplemental Table 3 - Skills and interventions	
**Skill: Team lead**	
Non-trauma resuscitation (MER)	19
Trauma resuscitation (TTA)	50
**Interventions**	
Advanced airway management in PED	by anesthesia by institutional default
Cardiopulmonary resuscitation	3
Central line placement	by PICU by institutional default
Chest drain insertion	by surgeon as default
Electric cardioversion	2
Inotropic support	by PICU by institutional default
Intraosseous line placement in PED	3
High-flow nasal cannula	12
Peripheral nerve block (femoral)	by anesthesia by institutional default
Pharmacologic cardioversion	8

TTA- trauma team activation, MER – medical emergency response, PED – pediatric emergency department, PICU – pediatric intensive care unit,
