# Supplementary material for: Critically Ill Children in a Swiss Pediatric Emergency Department With an Interdisciplinary Approach: A Prospective Cohort Study
Source: Front Pediatr. 2021 Oct 11;9:721646. doi: 10.3389/fped.2021.721646 (PMC8544259; doi:10.3389/fped.2021.721646)
Supplement: Supplementary file 4 [file Data_Sheet_4.pdf]

**Supplemental table 4 – PICU admission criteria**
**Airway/ Breathing**
All patients requiring mechanical ventilation
Patients with impending respiratory failure
Severe asthma requiring hourly inhalations
HFNC requiring more than 40% oxygen
**Circulation**
All patients after successful resuscitation (ROSC)
All types of shock/hemodynamic instability<br>- Hemorrhage<br>- Cardiogenic shock
Cardiac arrhythmias
**Neurologic**
Comatose patients
ICP Monitoring
**Metabolic**
Diabetic ketoacidosis pH <7.15
Severe electrolyte or acid base disturbances
Severe metabolic disorders
**Other**
Major trauma
Patients requiring hemofiltration
Patients requiring close monitoring due to potentially unstable conditions
PICU for stabilization and potentially transfer to dedicated national centers:<br>- CHD<br>- unstable transplant patient
ROSC - return of spontaneous circulation, ICP – intra cranial pressure, CHD – congenital heart disease, HFNC – high flow nasal cannula, PICU – pediatric intensive care unit
